# Meaning-Centered Psychotherapy for Cancer Caregivers: Study protocol of a randomized controlled trial

**DOI:** 10.1186/s13063-026-09621-7

**Published:** 2026-03-21

**Authors:** Allison J. Applebaum, Leah E. Walsh, Talia Zaider, Raymond E. Baser, Morgan J. Loschiavo, William S. Breitbart, Wendy G. Lichtenthal

**Affiliations:** 1 Brookdale Department of Geriatrics and Palliative Medicine, Icahn School of Medicine at Mount Sinai, New York, NY USA; 2 Department of Psychiatry and Behavioral Sciences, Memorial Sloan Kettering Cancer Center, New York, NY USA; 3 Department of Epidemiology and Biostatistics, Memorial Sloan Kettering Cancer Center, New York, NY USA; 4 Sylvester Comprehensive Cancer Center, University of Miami Health System, Miami, FL USA

**Keywords:** Cancer caregiver, Caregiver support, Meaning-Centered Psychotherapy for Cancer Caregivers, Meaning, Purpose, Spiritual wellbeing, Existential distress, Advanced cancer, Advance care planning, Bereavement

## Abstract

**Background:**

Caregivers of patients with advanced cancer shoulder immense responsibilities as they care for patients, including symptom and medication management, providing emotional support, and navigating healthcare treatment and decision-making. Due to the heavy toll of these responsibilities, caregivers are at high risk for profound mental health challenges, including anxiety, depression, and posttraumatic stress disorder. A key driver of this heightened risk for psychopathology in caregivers is existential distress, manifesting as a loss of meaning and purpose, decreased spiritual well-being, and hopelessness. Historically, psychosocial interventions targeting distress in cancer caregivers have neglected to address existential distress. Meaning-Centered Psychotherapy for Cancer Caregivers, a seven-session structured intervention, was developed to address this gap. In a pilot randomized controlled trial, the approach led to enhancements in personal meaning, benefit finding, and spiritual well-being. Here, we present a large, multi-site trial that aims to definitively examine the efficacy of Meaning-Centered Psychotherapy for Cancer Caregivers in an adequately powered study.

**Method:**

This randomized controlled trial will evaluate the efficacy of Meaning-Centered Psychotherapy for Cancer Caregivers versus Supportive Psychotherapy for Cancer Caregivers on primary (personal meaning and spiritual well-being) and secondary (anxiety, depression, sense of meaning in caregiving, benefit finding, caregiver burden, social support) outcomes at baseline, posttreatment, and at 6- and 12- months follow-up. It will also evaluate the role of sense of meaning in life as a mediator of secondary outcomes, as well as the impact of Meaning-Centered Psychotherapy for Cancer Caregivers on pre- and post-loss bereavement outcomes. Caregivers (*N* = 200) of patients with advanced (stage III/IV) solid tumor cancers from Memorial Sloan Kettering Cancer Center, Sylvester Comprehensive Cancer Center, and the community will be enrolled.

**Discussion:**

Meaning-Centered Psychotherapy for Cancer Caregivers has the potential to help alleviate existential suffering in caregivers as they manage the multifaceted demands of caring for patients with advanced cancer. This trial seeks to evaluate the efficacy of this intervention in a more robust and representative trial of cancer caregivers and extends prior research to explore mediators of improvement and the impact of the intervention on pre- and post-loss bereavement outcomes.

**Trial registration:**

ClinicalTrials.gov NCT06307535. Registered on 03/05/2024.

## Administrative information

Note: The numbers in curly brackets in this protocol refer to SPIRIT checklist item numbers. The order of the items has been modified to group similar items (see http://www.equator-network.org/reporting-guidelines/spirit-2013-statement-defining-standard-protocol-items-for-clinical-trials/).

**Table:** 

Title {1}	Meaning-Centered Psychotherapy for Cancer Caregivers (MCP-C): Study protocol of a randomized controlled trial
Trial registration {2a and 2b}	ClinicalTrials.gov NCT06307535, registered on 03/05/2024 https://clinicaltrials.gov/study/NCT06307535
Protocol version {3}	Icahn School of Medicine at Mount Sinai, July 22, 2025, version 1 (STUDY-24–01356).
Funding {4}	This study is supported by the National Cancer Institute (R01CA285621).
Author details {5a}	Allison J. Applebaum, PhD^1^ Leah E. Walsh, PhD^1^ Talia Zaider, PhD^2^ Raymond E. Baser^3^ Morgan Loschiavo^2^ William S. Breitbart^2^ Wendy G. Lichtenthal^4^ ^1^Brookdale Department of Geriatrics and Palliative Medicine, Icahn School of Medicine at Mount Sinai ^2^Department of Psychiatry and Behavioral Sciences, Memorial Sloan Kettering Cancer Center ^3^Department of Epidemiology and Biostatistics, Memorial Sloan Kettering Cancer Center ^4^Sylvester Comprehensive Cancer Center, University of Miami Health System
Name and contact information for the trial sponsor {5b}	This study is funded by the National Institutes of Health (R01CA28561), 9000 Rockville Pike, Bethesda, MD, 20892, USA
Role of sponsor {5c}	The trial sponsor had no role in the design of this study and will not have any role during its execution, analyses, interpretation of the data, writing of the report, or the decision to submit the report for publication.

## Introduction

### Background and rationale {6a}

Over 63 million individuals in the United States are caregivers, with a significant portion providing care to patients with life-limiting cancers [1]. Caregivers of patients with cancer take on tremendous responsibilities across the illness trajectory, assisting with symptom management [2], providing emotional support [3, 4], and navigating the healthcare team and treatment [5], all while managing other personal, family, occupational, and financial responsibilities. The burden of cancer caregiving can lead to increased levels of anxiety, depression [6], and posttraumatic stress [7]. Without timely intervention, such distress has the potential to negatively impact the care provided to patients [8–10] and can contribute to poor adjustment and psychosocial outcomes in bereavement [11, 12], such as prolonged grief disorder [13].

A key driver of these mental health outcomes is existential distress, characterized as a loss of meaning and purpose in life, decreased spiritual well-being, and hopelessness [14, 15]. Existential distress is common among caregivers due to the competing demands of caregiving and personal life goals and balancing hope for the present with an awareness of potential patient death in the future. Witnessing prolonged or aggressive medical treatment, patient suffering, or a decline in patient functioning can also contribute to existential distress [16–18]. Importantly, caregivers who experience existential distress are less likely to engage in advance care planning discussions with patients and healthcare professionals, which ultimately negatively impacts the care patients receive at the end of life [19]. As such, addressing existential distress in cancer caregivers has profound benefits for caregivers, as well as the patients for whom they provide care. When existential distress is mitigated, caregivers are better able to cultivate deeper connections with patients and strengthen their sense of meaning in caregiving. This in turn can reduce regret and unfinished business and prevent poor bereavement outcomes [20–22]. Despite these facts, existential distress has historically remained unaddressed in psychosocial interventions for cancer caregivers.

Meaning-Centered Psychotherapy (MCP) [23, 24] is an empirically supported intervention originally developed to help patients with advanced, life-limiting cancers connect to meaning and purpose, despite the challenges they face. Our team adapted MCP to assist cancer caregivers to connect to meaning and purpose and mitigate existential distress (Meaning-Centered Psychotherapy for Cancer Caregivers—MCP-C) [25, 26]. Developed with feedback from caregiver stakeholders, MCP-C is an existentially oriented seven-session manualized intervention that helps caregivers connect—or reconnect—to sources of meaning and purpose in their life despite the challenges, limitations, and losses that come with caregiving. Our pilot trial of MCP-C among 60 caregivers of patients with glioblastoma evidenced strong preliminary effects on enhanced personal meaning, benefit finding, and spiritual well-being. The results of this pilot randomized controlled trial, alongside qualitative feedback from participants that supported its feasibility and acceptability [27], suggested that MCP-C should be tested in a larger, fully powered randomized controlled trial.

## Objectives {7}

This randomized controlled trial has three objectives. The primary objective of this study is to investigate the efficacy of MCP-C versus Supportive Psychotherapy for Cancer Caregivers (SP-C) on primary (i.e., sense of meaning in life, spiritual well-being) and secondary (i.e., anxiety, depression, sense of meaning in caregiving, benefit finding, caregiver burden, social support) outcomes immediately posttreatment and explore maintenance in improvements up to 1-year posttreatment (Aim 1). The secondary objectives of this study are to evaluate the role of sense of meaning in life as a mediator of improvements in key secondary outcomes (Aim 2) and explore the effects of MCP-C on pre- and post-loss grief and bereavement outcomes (Aim 3).

## Trial design {8}

This is a randomized controlled trial in which participating caregivers (*N* = 200) are randomized 1:1 in a parallel group design to either MCP-C or SP-C to test the efficacy (superiority) of MCP-C versus SP-C.

## Methods: participants, interventions, and outcomes

### Study setting {9}

The coordinating site for this trial is the Icahn School of Medicine at Mount Sinai (ISMMS). Participants are being recruited from two primary sites: Memorial Sloan Kettering Cancer Center (MSK) and Sylvester Comprehensive Cancer Center (SCCC), part of the University of Miami Health System. Caregivers will also be recruited through the community via the National Alliance for Caregiving and the American Cancer Society, who will share information about this trial with their listservs and on their social media platforms. Information about study participation will be included on all advertisements and posts, and caregivers will be directed to contact ISMMS to learn more about study participation.

### Eligibility criteria {10}

Participants are eligible if they are as follows: At least 18 years of age, can read and understand English, a self-reported current caregiver to a patient with Stages III or IV solid tumor cancer currently receiving any type of medical treatment (e.g., curative, palliative), reporting caregiving-related distress as evidenced by scoring a 4 or greater on an adapted distress thermometer (DT) for caregivers, residing in New York, New Jersey, Florida, or Connecticut, or have the ability to complete sessions in compliance with current telehealth regulations. Participants are not eligible if they do not have a reasonable understanding of study procedures as judged by the consenting professional or if they are engaged in regular individual psychotherapeutic support that they are unable or unwilling to pause while receiving the study intervention.

MCP-C and SP-C interventionists will be eligible if they have, at minimum, a master’s degree and at least 1 year of prior clinical experience. They will be drawn from the disciplines of psychology, psychiatry, and social work with appropriate training and knowledge of individual therapeutic dynamics.

### Who will take informed consent? {26a}

Study team members trained in informed consent procedures will screen eligible participants, explain study procedures, and enroll caregivers who meet study inclusion criteria and provide informed consent. These consent conversations will occur over the telephone, and participants will self-report if they are able to comply with telehealth regulations throughout the MCP-C/SP-C sessions, that is, be physically located in states where interventionists are licensed during sessions.

### Additional consent provisions for collection and use of participant data and biological specimens {26b}

No ancillary studies or biological specimen collection is planned.

## Interventions

### Explanation for the choice of comparators {6b}

We are comparing MCP-C, the study intervention, to SP-C. SP-C is based on a model of supportive psychotherapy that has been used in previous randomized trials of MCP as the comparator intervention and was adapted to represent the standard of caregiver support in the community. It has also been standardized for use in RCTs [28].

### Intervention description {11a}

#### Meaning-Centered Psychotherapy for Cancer Caregivers (MCP-C)

MCP-C is a seven-session intervention delivered individually over telehealth platforms that combines didactics, discussion, and experiential exercises focused on themes related to meaning and caregiving. Each session is an hour long. Session 1 (*Concepts and Sources of Meaning*) provides an overview of meaning and caregiving and asks participants to share their caregiving journey and their understanding of the word “caregiver.” Session 2 (*Identity Before and After Becoming a Caregiver*) focuses on how caregiving impacts participants’ identities, highlighting both immutable aspects as well as the possibility for growth. Session 3 (*Historical Sources of Meaning*) focuses on the meaning derived through reflecting on one’s past legacy (i.e., family history and past life experiences that one does not choose), the present legacy caregivers are actively creating, and the future legacy they will give to others. Session 4 (*Attitudinal Sources of Meaning*) focuses on helping caregivers to recognize where they have choice and how meaning can be derived through reflecting on how they choose to respond to life’s limitations and challenges, including how they choose to face the potential eventual death of their care partner. Session 5 (*Creative Sources of Meaning*) focuses on meaning generated through creating one’s life (i.e., the activities they do or pursuits or interests that make them who they are) and highlights self-care as a key route through which caregivers can continue to create their lives, despite the challenges of caregiving. Session 6 (*Experiential Sources of Meaning*) focuses on meaning derived through experiencing life through the five senses and through the experiences of love, beauty, and humor. Session 7 (*Transitions*) reviews the material covered and provides space to reflect on the ways participants can continue to use these sources of meaning as resources moving forward. At the end of each session, caregivers are encouraged to reflect on questions specific to the subsequent session’s theme for homework to help guide discussion and reflection.

#### Supportive Psychotherapy for Cancer Caregivers (SP-C)

SP-C is a seven-session intervention delivered individually over telehealth platforms based on conceptual models developed by Rogers [29] and Block [30]. Each session is an hour long. SP-C incorporates client-centered, supportive strategies and offers caregivers a safe space to share challenges they face in their daily routines and experiences. For each session, the topic of discussion is determined by the caregiver and centers on their most pressing caregiving-related need at that time. Unlike in MCP-C, the therapist does not impose a structure or agenda onto the session. The role of the therapist is to provide supportive reflections, to acknowledge and validate their emotions and reactions in response to the caregiving role, to encourage caregivers’ self-reflection, and to highlight adaptive coping responses to challenges, including support-seeking and self-care strategies. Therapists utilize curiosity, acceptance, and openness to facilitate the therapeutic alliance to assist caregivers to explore and validate their experiences. SP-C has been utilized and standardized in past RCTs of MCP and tailored for cancer caregivers.

### Criteria for discontinuing or modifying allocated interventions {11b}

Discontinuing the allocated intervention will only occur if the participant chooses to end their participation in the study or is no longer able to comply with telehealth regulations. Participants will not be able to modify their allocated intervention arm once randomized. There are no significant concerns of harm from either study arm that would require a change in study arm allocation. If participants become bereaved, they are welcome—and encouraged—to remain on the study, and bereavement-related adaptations are made to subsequent sessions.

### Strategies to improve adherence to interventions {11c}

We encourage participants to complete study sessions weekly and will be flexible with their schedule if they need to reschedule a session for a later date. We expect that the seven study sessions will be completed within 14 weeks. This schedule flexibility will help to improve adherence to the intervention. All intervention sessions are audio/video recorded, and a random sample of 30% of cases (100% of sessions for these participants) will be evaluated and rated for treatment integrity by independent raters throughout the study. We have developed comprehensive treatment manuals for MCP-C and SP-C to facilitate standardized delivery, and all interventionists undergo a half-day training in either MCP-C or SP-C, followed by weekly group supervision sessions. Supervisors also review recorded sessions to facilitate reinforcement of model-specific strategies and alert therapists of any drift in model adherence. To reduce likelihood of treatment contamination, each condition is delivered by distinct treatment providers. Further, therapists monitor attendance and homework completion when relevant as part of session notes. Deviations from the protocol are recorded and discussed regularly during weekly group supervision.

### Relevant concomitant care permitted or prohibited during the trial {11d}

Caregivers will be ineligible for participation if they are engaged in regular individual psychotherapeutic support that they are unable or unwilling to put on hold for the course of the intervention. The use of psychotropic medications while engaging in the trial is permitted.

### Provisions for posttrial care {30}

There are no provisions for posttrial care. However, should a participant specifically request referrals for ongoing support after completion of the trial, the study team will provide appropriate resources.

### Outcomes {12}

**Fig. 1:**
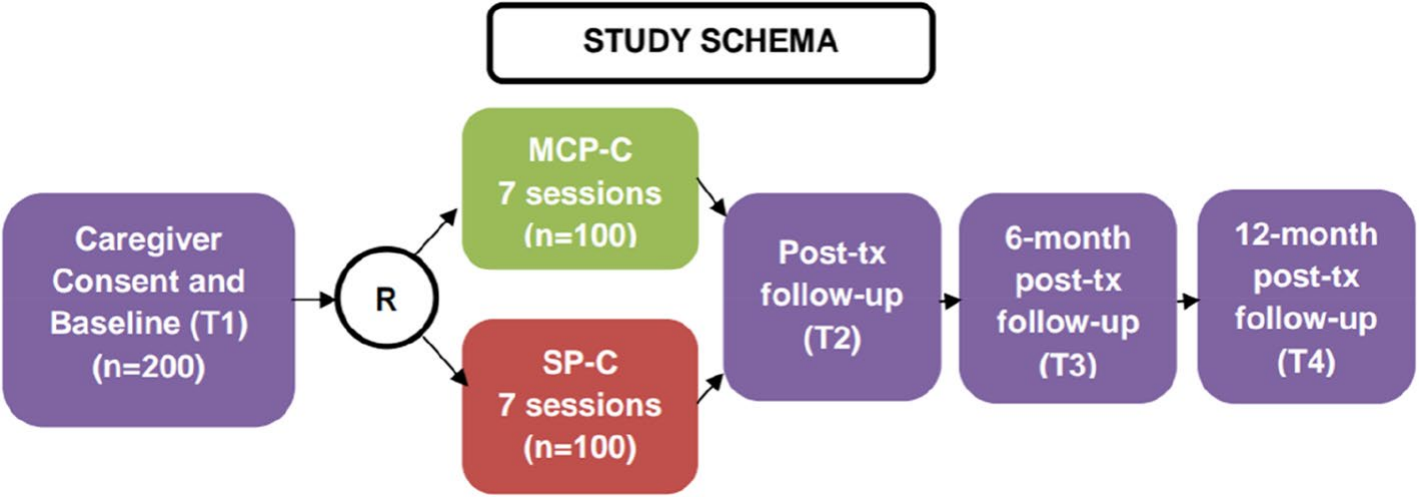
Study timeline

**Fig. 2:**
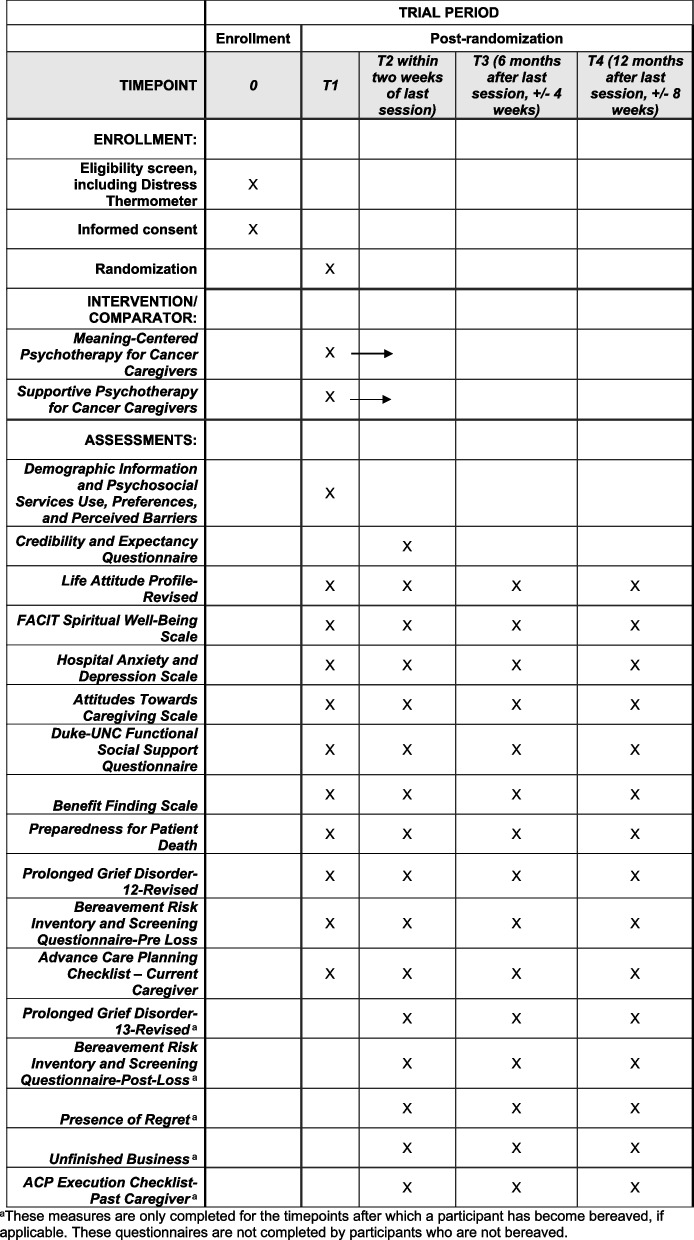
SPIRIT figure. Participant timeline: schedule of enrollment, interventions, and assessments. ^a^This measure is only completed for the time points after which a participant has become bereaved, if applicable. These questionnaires are not completed by participants who are not bereaved

As outlined in the participant timeline, assessments will occur at four time points: baseline (T1), posttreatment (T2), and 6-month (T3) and 12-month (T4) follow-up (Fig. 1). Section “Plans for assessment and collection of outcomes {18a}” includes detailed descriptions of outcome measures. The SPIRIT figure for the trial is shown in Fig. 2.

All primary and secondary outcome measures for Aim 1 are continuous variables that will be assessed at all four time points (T1, T2, T3, and T4). For each outcome measure, our main parameter of interest is the model-estimated difference in mean change scores from T1 to T2 between the MCP-C and SP-C treatment arms (see section {20a} for model details). For each outcome, as exploratory parameters, we will also examine the model-estimated differences in mean change scores from T1 to T3 and from T1 to T4 between the MCP-C and SP-C treatment arms. The two co-primary outcome measures are sense of meaning in life, as measured by the LAP-R PMI, and spiritual well-being, as measured by the FACIT SWBS. Secondary outcome measures include anxiety (HADS-A subscale) and depression (HADS-D subscale), sense of meaning and purpose in caregiving (ATCS total score), benefit finding (BFS), caregiver burden (CRA), and social support (FSSQ).

For Aim 2, we will evaluate the extent to which changes in LAP-R PMI scores from T1 to T2 mediate the model-estimated differences in mean change scores from T1 to T3 of three outcome measures (BFS, HADS-A, and HADS-D) between the MCP-C and SP-C treatment arms.

The Aim 3 bereavement outcomes are divided into pre-loss and post-loss outcome measures. The pre-loss measures will be administered at T1 to all caregivers and at T2, T3, and T4 for caregivers who remain non-bereaved at those time points. The post-loss measures will be administered at T2, T3, and T4 to caregivers who are bereaved at those time points. Because of the reduced sample sizes for the analyses of the pre-loss and post-loss outcomes, the Aim 3 outcomes are exploratory. The pre-loss outcomes are preparedness for patient death (three-category response to single item with values “not at all,” “somewhat,” and “very”), pre-loss grief intensity (PG-12-R score), and risk for bereavement mental health challenges (BRISQ-P score). For preparedness for patient death, the parameters of interest are differences between the two treatment arms in the model-estimated proportions of non-bereaved caregivers responding “somewhat” or “very” at T4. For the PG-12-R and BRISQ-P, our main parameter of interest is the model-estimated difference in mean change scores from T1 to T2 between the two treatment arms among non-bereaved caregivers. The post-loss outcomes include two dichotomous (yes/no) outcomes (the presence of regret, unfinished business) and the continuous outcomes of post-loss grief intensity (PG-13-R) and risk for post-loss bereavement mental health challenges (BRISQ-B). The parameters of interest for the presence of regret and unfinished business are the differences between the treatment arms in the model-estimated proportions of bereaved caregivers responding “yes” to these items at T4. For the PG-13-R and BRISQ-B, our main parameter of interest is the model-estimated difference in mean scores at T4 between the two treatment arms among bereaved caregivers.

### Participant timeline {13}

Potential participants will be screened for eligibility (approximately 5 min) and will meet criteria if they report distress ≥ 4 on the distress thermometer and indicate that the distress is related to caregiving. If interested, participants will complete informed consent procedures and will be offered to immediately complete baseline (T1) assessments. Participants will have up to 45 days (consent expiration) to complete baseline assessments. Once baseline assessments are complete, they will be randomized to either MCP-C or SP-C and assigned to a therapist for seven individual sessions that will be completed over Webex. Participants will have up to 14 weeks to complete all seven sessions. After intervention completion, participants will complete follow-up assessments immediately posttreatment (T2, +2 weeks), 6-month follow-up (T3, ±4 weeks), and 12-month follow-up (T4, ±8 weeks). See Fig. 1 for the full study timeline.

### Sample size {14}

Participants will be 200 caregivers recruited across study sites and the community, with 100 caregivers randomized to each study arm (MCP-C vs. SP-C). The sample size was chosen to provide 80% power to detect reasonable treatment effect sizes for our two primary endpoints while maintaining the type 1 error rate at *α*= 0.05 for these comparisons. Using methods relevant to clinical trials with repeated measures [31], we calculated the smallest effect size that we will be able to detect for the immediate posttreatment coefficient of the time-by-arm interaction term in our linear mixed model analysis of one of the two primary outcome measures (LAP-R PMI). Given the sample size (*N* = 200), we will be able to detect a standardized mean difference treatment effect size between the two study arms of 0.48 standard deviations. This calculation assumed a correlation between baseline and follow-up measures of 0.5, 20% attrition by the posttreatment assessment, a two-sided significance test, and a type 1 error of 0.025 (0.05 overall for both primary endpoints).

### Recruitment {15}

To maximize recruitment and retention, this study employs a multi-site design where participants will be recruited from outpatient clinics in various departments at MSK and SCCC. At both sites, we will employ three recruitment strategies. Flyers will be posted (digitally or using paper with the clinic’s permission) in clinics at MSK and SCCC. Study contact information will be provided on each flyer where caregivers can reach out to learn more about the study. Participants will also be recruited through patient appointments. Physicians will either directly provide caregivers with the contact information of the study team, or the study team will approach caregivers during or after patients’ next clinic appointment and introduce the study. Third, if clinicians who are aware of the study receive an inquiry from a caregiver about supportive services, they will provide their information to the study team who will subsequently send a study invitation letter to the caregiver via email. The study team will then make 3–5 follow-up calls/emails to discuss interest and participation with the caregiver.

Recruitment from community partners will occur through listservs and social media channels (e.g., Twitter/X, Facebook) of both the American Cancer Society and the National Alliance for Caregiving in states where MCP-C and SP-C supervisors are licensed (New York, New Jersey, Florida, and Connecticut). These advertisements will include study team information and will direct potential participants to contact the study team by email. Caregivers from New York and New Jersey will be enrolled at MSK, and those from Florida and Connecticut will be enrolled at SCCC. During the initial approach and informed consent conversation, study team members will ensure that participants reside in a state where the MCP-C and SP-C supervisors are licensed and may be asked to provide certain health information that is necessary for the recruitment and enrollment process.

## Assignment of interventions: allocation

### Sequence generation {16a}

Participants will be randomized to either MCP-C or SP-C using MSK’s clinical research database, a secure computer system that ensures allocation concealment. Randomization will be 1:1 (MCP-C:SP-C) stratified by study site (MSK, SCCC, community/MSK, and community/SCCC) using randomly permuted blocks of random length. Given the clear focus of MCP-C versus SP-C on unique meaning-focused themes, participants and their study interventionists will not be blinded to intervention assignment.

### Concealment mechanism {16b}

#### Implementation {16c}

Participants will be randomized 1:1, stratified by study site, to MCP-C or SP-C using MSK’s clinical research database, a secure computer system that generates the randomization sequence and ensures allocation concealment by using randomly permuted blocks of random length to allocate participants to study arm. Participants will be enrolled by a consenting professional at the corresponding study site, and the clinical research database will assign the intervention arm.

## Assignment of interventions: blinding

### Who will be blinded {17a}

Neither participants nor their study interventionists delivering MCP-C and SP-C will be blinded to intervention assignment due to the clear focus of the MCP-C intervention on meaning and existential content and SP-C on the provision of support. However, the study biostatistician who will conduct the analyses will be blinded.

### Procedure for unblinding if needed {17b}

N/A.

## Data collection and management

### Plans for assessment and collection of outcomes {18a}

Data will be collected via self-report measures using the Research Electronic Data Capture (REDCap), a secure web application for creating and managing online survey databases. The Clinical Research Coordinator (CRC)—who receives training in data quality assurance—will send all study assessments to participants at the appropriate timepoint. Outcomes will be measured at four time points: baseline, directly after informed consent or until consent expiration (i.e., 45 days; T1), immediately post-intervention + 2 weeks (T2), 6-month follow-up ± 4 weeks (T3), and 12-month follow-up ± 8 weeks (T4). This will allow flexibility for study assessment completion and aid caregiver retention throughout the entire study. All data collection forms can be found in the study protocol.

At baseline (T1), participants will complete demographic information (e.g., age, sex, race, ethnicity, income), caregiver characteristics (e.g., relationship to the patient, length of caregiving), and a survey about psychosocial service use, preferences, and perceived barriers, using established questionnaires from our group’s previous studies. Participants will also complete all outcome measures (i.e., sense of meaning in life, spiritual well-being, anxiety and depression, benefit finding, meaning in caregiving, caregiver burden, and social support) at T1. At T2, in addition to study outcome measures, participants will complete the Credibility and Expectancy Questionnaire (CEQ) to assess treatment acceptability and perceived effectiveness. Pre-loss (preparedness for patient death, pre-loss grief intensity, risk for bereavement mental health challenges, engagement in advance care planning) and post-loss (the presence of regret, unfinished business, post-loss grief intensity, risk for post-loss bereavement mental health challenges, and occurrence of advance care planning discussions and goal-concordant care at patients’ end of life) bereavement outcomes will also be explored at all timepoints.

#### Description of study measures

##### Screening and non-outcome measures

- Caregiving-related distress: A modified version of the Distress Thermometer (DT) [32] will be used to screen for distress related to caregiving. Participants are asked to rate their distress on a scale of 0–10 (cutoff score ≥ 4) and indicate that their distress is related to caregiving to be eligible for the study. The distress thermometer will be administered at screening only.
- Treatment acceptability and perceived effectiveness: The Credibility and Expectancy Questionnaire (CEQ) is a 6-item measure of treatment acceptability and perceived effectiveness [33, 34]. This measure has been used extensively in prior research and has strong psychometric properties [34]. The CEQ is administered at T2 only.

##### Primary outcomes

- Sense of meaning in life: The Life Attitudes Profile-Revised (LAP-R) [35] is a 48-item measure of meaning in life, with items rated on a 7-point Likert scale from *strongly agree* to *strongly disagree*, with higher scores indicating a stronger presence of that type of meaning. The LAP-R generates two composite scores: the Personal Meaning Index (PMI) and existential transcendence (ET). The LAP-R has demonstrated strong internal consistency in previous research (Cronbach’s α range: 0.77–0.91) [35]. The LAP-R is administered at T1–T4. The LAP-R PMI at T2 is one of our two co-primary outcome measures.
- Spiritual wellbeing: The FACIT Spiritual Well-Being Scale (SWBS) [36] is a 12-item measure of spiritual well-being measured on a 0 (not at all) to 4 (very much) scale, with higher scores indicating higher spiritual wellbeing. This measure has demonstrated strong internal consistency across all subscales in cancer caregivers (Cronbach’s α range: 0.88–0.93) [26]. The SWBS is administered at T1–T4. The FACIT SWBS at T2 is one of our two co-primary outcome measures.

##### Secondary outcomes

- Anxiety and depressive symptoms: The Hospital Anxiety and Depression Scale (HADS) is a 14-item measure of anxiety and depressive symptoms with items rated on a 0 to 3 scale, with higher scores indicating greater symptomatology. Scoring the HADS yields anxiety (HADS-A) and depression (HADS-D) subscale scores, each consisting of seven items. The HADS subscales have demonstrated strong internal consistency in cancer caregiver populations (Cronbach’s α range: 0.82–0.92) [26, 37]. The HADS is administered at T1–T4.
- Sense of meaning and purpose in caregiving: The Attitudes Toward Caregiving Scale (ATCS) [38] is a 43-item measure rated on a 5-point Likert-type scale that examines the extent to which caregivers find meaning in caregiving. Scores can be summed for a total score in addition to three subscales: loss/powerlessness, provisional meaning, and ultimate meaning, with higher scores indicating higher overall meaning and purpose. The ATCS is administered at T1–T4 in non-bereaved caregivers.
- Benefit finding: The Benefit Finding Scale (BFS) is a 17-item measure of perceived benefit finding that has been modified for caregiver populations. It has been used in our group’s previous research and has demonstrated strong internal consistency (Cronbach’s α range: 0.91 to 0.95 [39, 40]. The BFS is administered at T1–T4.
- Caregiver burden: The Caregiver Reaction Assessment (CRA) is a 24-item measure with items scored on a 5-point Likert-type scale [41]. Items are summed for a total score (range: 24–120) across five subscales: self-esteem, family support, impact on finances, schedule, and health. The CRA is a widely used measure of caregiver burden that has demonstrated good internal consistency and construct validity [42]. The CRA is administered at T1–T4 in non-bereaved caregivers.
- Social support: The Duke-UNC Functional Social Support Questionnaire (FSSQ) is an 8-item measure of perceived social support. Items are summed to a total score ranging from 0 to 40, with higher scores indicating greater perceived social support. The FSSQ has demonstrated strong internal consistency (Cronbach’s α = 0.94) [43]. The FSSQ will be administered at T1–T4.

## Bereavement-related outcomes

### Pre-loss outcomes

- Preparedness for patient death: The preparedness for patient death item asks participants “If your care partner were to die soon, how prepared would you be for his/her death?” Response options are “not at all,” “somewhat,” and “very.” This item is based on previous studies of preparedness for loss [44–46]. Preparedness for patient death is measured at T1–T4 in non-bereaved caregivers.
- Pre-loss grief intensity: The Prolonged Grief-12-Revised (PG-12-R) is a 12-item measure of pre-loss grief, adapted from a measure of prolonged grief symptoms [44] that is worded to reflect the patient as living. PG-12-R items are rated on a 5-point Likert-type scale, with total scores ranging from 11 to 55 and with higher scores suggesting greater anticipatory grief. The PG-12-R has demonstrated strong internal consistency [47] and is administered at T1–T4 to non-bereaved caregivers.
- Risk for bereavement-related mental health challenges: The Bereavement Risk Inventory and Screening Questionnaire-Pre-Loss (BRISQ-P) is a 30-item screener for bereavement mental health challenges, with items rated on a 5-point Likert scale [48]. The BRISQ-P assesses illness or death-related and bereavement-related risk factors and is administered at T1–T4 to non-bereaved caregivers.
- Advance care planning engagement: Engagement in advance care planning will be measured by the Advance Care Planning (ACP) Checklist–Current Caregiver [49] which includes 14 items that ask caregivers about previous ACP discussions and domains of ACP (e.g., prognosis, goals of care, execution of advance directives). Items are scored and summed to a total score. This checklist is administered at T1–T4 to non-bereaved caregivers.

### Post-loss outcomes

- Post-loss grief intensity: The Prolonged Grief-13-Revised (PG-13-R) is a 13-item measure that evaluates symptoms of prolonged grief disorder [50]. It assesses 10 grief-related symptoms on a 5-point Likert scale. Scores range from 10 to 50 with higher scores indicating greater grief intensity. The PG-13-R is administered at T2–T4 to caregivers who become bereaved during the study.
- Risk for bereavement-related mental health challenges post-death: The Bereavement Risk Inventory and Screening Questionnaire-Post-Loss (BRISQ-B) is a measure of risk for bereavement mental health challenges following the patient’s death [48]. It is nearly identical to the BRISQ-P, but items are modified to refer to the patient’s death rather than their illness. It also includes items related to the bereavement experience. Items are rated on a 5-point Likert-type scale. The BRISQ-B is administered at T2–T4 to caregivers who become bereaved during the study period.
- The Presence of Regret: The single Presence of Regret item [22] asks caregivers, “Do you regret any of your actions or choices that occurred while providing care to your loved one with cancer when they were ill?” with a dichotomous yes/no response option. For those who report yes, they will be asked to provide an example of their most troubling regret and to rate their distress about the stated regret on a 10-point scale (10 = most distressed). The Presence of Regret item will be administered at T2–T4 for those caregivers who become bereaved during the study period.
- Unfinished business: The single unfinished business item asks caregivers, “Do you feel that anything was unfinished, unsaid, or unresolved in your relationship with your loved one with cancer?” with a yes/no response option. If yes, caregivers are asked to provide an example of their most troubling source of unfinished business and to rate their distress about this on a 10-point scale (10 = most distressed). This item was developed to be face valid and consistent with theory on regret and unfinished business [20, 20, 51] and has demonstrated good concurrent validity [21].
- Advance care planning engagement: Caregivers who become bereaved will complete the Advance Care Planning Checklist–Past Caregiver, which mirrors the Advance Care Planning Checklist–Current Caregiver but asks about what type of care the patient received at end of life, where the patient died, and if the location of death and care provided were consistent with the patient’s stated wishes.

### Plans to promote participant retention and complete follow-up {18b}

Participants will be compensated $10 for each intervention session they complete. They will also be compensated $5 for completing baseline assessments (T1), $10 for completing post-intervention assessments (T2), $15 for completing 6-month follow-up assessments (T3), and $20 for completing 12-month follow-up assessments (T4). Participants are sent a maximum of three emails and called a maximum of three times to remind them to complete each survey. Those participants who discontinue their involvement in the study intervention will be asked to continue to complete study assessments and will receive reimbursement for completed assessments. Participants enrolled through MSK will receive their incentives via Amazon gift card codes and those enrolled through SCCC, via ClinCard.

### Data management {19}

A Clinical Research Coordinator (CRC) is assigned to this study whose responsibilities include project management and compliance, data collection, data entry, data reporting, regulatory monitoring, identifying and resolving problems, and coordinating activities related to the study protocol. The maintained dataset will include minimal data (race, ethnicity, sex, and age) and will be entered into the Clinical Research Administration Clinical Trials Management System.

Data collected for this study will be entered into and managed via the secure Research Electronic Data Capture (REDCap) database. REDCap is an open-source platform that allows for data collection in a secure manner over a web-based interface. The REDCap platform was developed by Vanderbilt University, with whom ISMMS has a standing agreement to allow the usage of REDCap for academic/research purposes. REDCap prevents users from entering data outside of predetermined ranges of possible scores. Participants complete all assessments directly in REDCap, eliminating the need for double data entry of paper assessment forms.

To ensure data quality, reports will be generated periodically to monitor accrual and completeness of participant registration data. Data quality reports will also be generated to assess missing data and inconsistencies. Throughout the study, data on accrual rates and follow-up will be monitored to identify any potential problems that will be brought to the attention of the study team during weekly team meetings. An audit of consent and eligibility data will be performed with the first five enrolled participants to assess for data completeness and data accuracy.

### Confidentiality {27}

Research staff involved in participant recruitment will provide an overview of the study, invite potential participants to participate, and discuss potential risks and benefits during the informed consent process. All participants are required to provide informed consent prior to completing study assessments and the intervention. Participants can choose to skip any question in the assessment battery they do not wish to answer. Participants who report experiencing psychological distress related to completing study questions will be offered support from the enrolling institutions’ counseling or supportive care services or receive community referrals for mental health providers.

When presenting study results, data will be presented for the entire sample and will not reference any individual participant’s data. All survey data will be de-identified using a code number to ensure confidentiality of participant data. For research staff who need to access study data, password security measures and other relevant restrictions will be applied to maintain confidentiality. Confidential data will only be accessible on a “need-to-know” basis for any particular research staff member. All personnel involved with the study will receive training in HIPAA and human subjects’ protections and will receive information on the ethics of accessing electronic data.

### Plans for collection, laboratory evaluation, and storage of biological specimens for genetic or molecular analysis in this trial/future use {33}

No biospecimen data will be collected as part of the study aims and procedures.

## Statistical methods

### Statistical methods for primary and secondary outcomes {20a}

Descriptive statistics will be calculated for baseline demographic and caregiver clinical variables, summarizing each outcome by treatment arm. We will assess the distribution of each study outcome and, if necessary, perform corrective transformations before calculating inferential statistics. We will also perform all efficacy analyses by the intention-to-treat principle, meaning that all randomized participants will be analyzed regardless of dropout or treatment adherence in their respective study arm. All endpoint comparisons between study arms will be adjusted for the randomization stratification variable (study site).

#### Aim 1: Analytic plan

The primary and secondary outcome measures for Aim 1 are all continuous variables assessed repeatedly over the four time points: baseline (T1), immediately posttreatment (T2), 6-month follow-up (T3), and 12-month follow-up (T4). The co-primary efficacy endpoints for Aim 1 are the LAP-R PMI and FACIT SWBS at the immediately posttreatment assessment (T2). We will consider MCP-C efficacious if MCP-C participants have significantly greater improvements than SP-C participants in LAP-R PMI or FACIT SWBS scores at T2 at the *p*< 0.025 statistical significance threshold in the analysis described below. We will analyze each continuous primary and secondary outcome measure by using constrained linear mixed model (cLMM) regression [52–54]. This statistical method accounts for within-subject correlations due to repeated measurement of participants and, under the reasonable missing at random (MAR) assumption, allows unbiased estimation of between-group differences without excluding participants with missing follow-up data. Each of these outcome measures will be analyzed using a separate cLMM regression with random intercept that will model the baseline and repeated follow-up scores (dependent variable) as a function of time (categorical) and the time-by-arm interaction, controlling for the randomization stratification variable (study site). The cLMM method will also constrain the two study arms to have a common baseline mean on the outcome measure to reflect the pre-randomization nature of the baseline assessment (T1) [52]. All randomized participants will have, at a minimum, the baseline (T1) primary outcome assessments and will be included in the cLMM per the intention-to-treat principle. In the cLMM parameterization, the coefficients for the time-by-arm interaction are equivalent to the differences between arms in outcome measure score changes from baseline to the post-baseline time points, and they will be calculated with 95% confidence intervals (CIs). For each co-primary endpoint (LAP-R PMI and FACIT SWBS), we will conclude that MCP-C participants had significantly larger improvements in the endpoint at T2 compared to SP-C if the time-by-arm interaction coefficient for T2 is significantly larger than 0 at the *p* < 0.025 level. The secondary outcome measures will be compared between study arms using similar cLMM analyses. However, we will assess estimated treatment effects at each timepoint using 95% CIs instead of *p*-values.

#### Aim 2: Analytic plan

We will use model-based causal mediation analysis (CMA) [55] to evaluate whether changes in sense of meaning in life (LAP-R PMI) immediately posttreatment (T2) mediate the effect of treatment arm on benefit finding (BFS), anxiety (HADS-A), and depression (HADS-D) at 6-month follow-up. We will use the *mediation* [56] package in R [57] to conduct mediation analyses for each of the three outcomes (BFS, HADS-A, HADS-D). Each mediation analysis will estimate the average causal mediation effect (ACME, the effect of the treatment arm on the outcome via the mediator), average direct effect (ADE, effect of treatment arm on the outcome measure excluding the mediation), the average total effect (ATE, sum of the ACME and ATE), and the proportion of the ATE mediated by the mediator variable (ACME/ATE). To estimate these effects, we will input two models, (1) a mediator model and (2) an outcome model, to the estimation functions in the *mediation*R package. The (1) mediator model will be a linear regression with T2 LAP-R PMI as the dependent variable and treatment arm and baseline (T1) LAP-R PMI as independent variables, controlling for baseline outcome measure (e.g., T1 BFS) and study site (randomization stratification variable). The (2) outcome model will be a linear regression with the T3 outcome measure (e.g., BFS) as the dependent variable and treatment arm, baseline outcome measure, T2 and T1 LAP-R PMI, and study site (randomization stratification variable) as independent variables. We will conduct these mediation analyses once excluding those participants with missing data for any variables in the model, and we will conduct sensitivity analyses replicating the model after applying multiple imputation of missing data using chained equation analyses [58]. Other sensitivity analyses will be conducted to evaluate results testing the impact of unmeasured pre-treatment confounding variables [59].

#### Aim 3: Analytic plan

Based on our group’s prior research, we estimate that approximately 50 (25%) participants in this study will become bereaved by the 12-month follow-up assessment (T4). Among the subset of non-bereaved caregivers, we will utilize the same cLMM analysis used in assessing the secondary outcomes in Aim 1 to examine differences between study arms in risk for bereavement mental health challenges (BRISQ-P) and pre-loss grief (PG-12-R). Multinomial logistic regression analyses will assess between-arm differences in T4 preparedness for loss in this subset, controlling for study site and baseline preparedness for loss. Among the subset of caregivers who become bereaved by T4, we will assess between-arm differences in T4 risk for bereavement mental health challenges (BRISQ-B), post-loss grief (PG-13-R), and the presence of regret (single item, yes/no) using linear (BRISQ-B, PG-13-R) and logistic (presence of regret) regression models controlling for study site and time since bereavement. Also, among those bereaved before T4, we will test whether higher posttreatment (T2) preparedness for patient death and/or engagement in ACP discussions (ACP Execution Checklist score) are associated with lower levels of T4 post-loss grief (PG-13-R) by fitting linear regression models predicting T4 PG-13-R scores. These models will control for study site, time since bereavement, and treatment arm, and they will include either T2 preparedness for patient death or the ACP Execution Checklist score as the focal predictor.

### Interim analyses {21b}

Not applicable.

### Methods for additional analyses (e.g., subgroup analyses) {20b}

All detailed analyses comparing study arms (MCP-C versus SP-C) will be adjusted for randomization stratification variable (study site).

### Methods in analysis to handle protocol nonadherence and any statistical methods to handle missing data {20c}

Efforts to minimize missing data include ensuring an efficient and organized trial design, robust training of research staff, and reducing participant burden by utilizing remote study assessments rather than in-person visits. For participants who report time constraints or difficulty completing study assessments, we will ask them to only complete primary outcome measures (LAP-R and FACIT SWBS) in order to minimize missing data regarding primary study aims. Missing data will be treated using sensitivity analyses and other data analytic approaches that are responsive to missingness. Specifically, we will first examine whether data missingness is associated with variables such as randomization arm and baseline outcome measures by comparing caregivers with complete data to those with missing data. If data is not missing at random, then we will modify analyses by controlling for factors associated with data missingness [60, 61].

### Plans to give access to the full protocol, participant-level data, and statistical code {31c}

Reasonable requests for the study protocol or de-identified data can be made to the principal investigator.

## Oversight and monitoring

### Composition of the coordinating center and trial steering committee {5d}

The study team at the coordinating center, ISMMS, is comprised of the CRC and study principal investigator. Co-investigators are housed at MSK and SCCC. The study team at the coordinating site meets weekly to discuss recruitment, active participants, and study questionnaire completion updates. There is no trial steering committee for the current study.

### Composition of the data monitoring committee and its role and reporting structure {21a}

ISMMS/Tisch Cancer Institute (TCI) is serving as the IRB of record, and as such, the data and safety monitoring plan is standard to clinical trials completed at TCI. The TCI Data and Safety Monitoring (DSM) plan ensures that all clinical trials are high quality, routinely monitored, and meet sponsor, institutional, and governmental requirements. The plan includes all required elements of the DSM as defined by the NIH and the NCI: monitoring responsibilities, description of what is to be monitored (e.g., data types), frequency of reviews, protocol compliance auditing procedures, adverse event reporting procedures, suspensions of clinical research, and conflicts of interest.

A formal Data and Safety Monitoring Committee (DSMC), independent of the sponsor, was established in 2008 to ensure that all clinical trials that are greater than minimal risk at TCI have adequate data and safety monitoring plans. Interventional and institutional studies, Investigator-Initiated Trials (IIT) requesting that the TCI DSMC serve as the independent Data and Safety Monitoring Board—as is the case with the current investigation—undergo review of the protocol and Data and Safety Monitoring Charter prior to review by the Protocol Review and Monitoring Committee (PRMC) review. The PRMC is charged with the review of all new cancer-related clinical research protocols at Mount Sinai to assess their scientific merit and prioritize them relative to the mission and scope of the TCI. The PRMC coordinator submits the protocol and charter to the DSMC for pre-review. The DSMC Chair, in coordination with the DSMC Administrator, assigns each initial review to a physician and a biostatistician based on the member’s disease specialty and accounting for conflicts of interest. The protocol is submitted before a monthly DSMC meeting, and questions for the PI and responses are documented in minutes. Protocol review includes a plan of review for each arm, dose level or stratum including the number of participants, predefined toxicities, dose adjustments, reporting structures for unanticipated problems and serious adverse events, response assessments, timing of reviews, dose-limiting toxicities, dose escalation rules, stopping criteria of the trial for toxicity or efficacy, and criteria defining maximum tolerated dose. Approval of the monitoring plan and charter is required before PRMC approval and is reviewed annually.

Further details are available in the TCI DSMC Charter.

### Adverse event reporting and harms {22}

There are no physical risks or side effects associated with participation in this study. Psychological risks are limited to possible distress related to engaging in a psychotherapy focused on the existential distress that arises from taking care of a patient with an advanced cancer. We will inform potential participants that it is reasonable not to participate if they feel discomfort in completing the assessment questionnaires or psychotherapy sessions. We will present questionnaires in a sequence that is sensitive to their recollection and thought about their experience of caregiving. All research staff will be trained to identify signs of psychological discomfort in participants, and participants reporting significant distress, including moderate depressive symptomatology (identified via the Hospital Anxiety and Depression Scale (HADS)) or suicidal ideation, will be referred to the in-house licensed clinical psychologist at MSK (Dr. Zaider) and SCCC (Dr. Lichtenthal) or covering psychologist or psychiatrist on call, who will provide immediate evaluation, support, and/or referral as needed.

This is a minimal risk study, and we will only report serious adverse events (SAEs) including deaths that are believed to be at least possibly related to the study intervention or participation. An adverse event is considered any of the following: death, a life-threatening adverse event, an adverse event that results in inpatient hospitalization or prolongation of an existing hospitalization, a persistent or significant incapacity or disruption to the ability to conduct normal life functions, a congenital anomaly/birth defect, and other important medical events that may not result in the above events but may jeopardize the patient or participant based on medical judgment and may require medical or surgical intervention to prevent one of the outcomes mentioned above.

### Frequency and plans for auditing trial conduct {23}

Quality assurance reports will be generated by the study team throughout the study period to monitor participant accrual and completeness of participant registration data. Data quality reports will also be generated to assess data missingness or inconsistencies. Data from the first five participants will be additionally audited for data and consent/eligibility completeness, accuracy, and psychological symptom levels. Thereafter, random samples of data will be assessed for quality and protocol compliance.

### Plans for communicating important protocol amendments to relevant parties (e.g. trial participants, ethical committees) {25}

All protocol amendments are reviewed by study investigators and approved by the appropriate Institutional Review Board ethics committees at each study site.

## Dissemination plans {31a}

Data from the current trial will be disseminated through peer-reviewed journal articles.

## Discussion

This trial builds on a previous pilot trial of MCP-C in caregivers of patients with glioblastoma multiforme that found preliminary evidence of improvements in meaning and purpose, spiritual well-being, and benefit finding. Here, this study protocol describes a fully powered randomized controlled trial to more comprehensively establish the efficacy of MCP-C versus SP-C on enhancing meaning, spiritual well-being, anxiety, depression, benefit finding, and social support, as well as on pre- and post-loss bereavement-related outcomes. Findings from this study will contribute to knowledge on the effects of MCP-C on existential distress, spirituality, and other psychosocial outcomes. It will also extend the psychosocial oncology and caregiving science literature to better understand the impact of MCP-C on bereavement outcomes in a diverse sample of caregivers of patients with advanced, solid-tumor cancers.

## Trial status

This trial (protocol version 5) is currently recruiting and enrolling caregivers, collecting data, delivering the interventions, and collecting follow-up data. Recruitment began on March 13, 2024, and the first participant was consented on that same day. We anticipate completing recruitment by March 2028.

## Data Availability

Data and materials are available upon reasonable request and agreement from the study PI. The study PI and biostatistician will have access to the final trial dataset.

## Abbreviations

ACP: Advance care planning
ATCS: Attitudes Toward Caregiving Scale
BFS: Benefit Finding Scale
CRA: Caregiver reaction assessment
CRC: Clinical research coordinator
FACIT: Functional Assessment of Chronic Illness Therapy
FSSQ: Duke-UNC Functional Social Support Questionnaire
HADS: Hospital Anxiety and Depression Scale
ISMMS: Icahn School of Medicine at Mount Sinai
LAP-R PMI: Life Attitude Profile-Revised, Personal Meaning Index
MCP-C: Meaning Centered Psychotherapy for Cancer Caregivers
MSK: Memorial Sloan Kettering Cancer Center
SAE: Serious adverse events
SCCC: Sylvester Comprehensive Cancer Center
SP-C: Supportive Psychotherapy for Cancer Caregivers
SWBS: FACIT Spiritual Well-Being Scale
